# Incidental pulmonary embolism and the value of CT pulmonary angiography follow-up versus expert review

**DOI:** 10.1093/bjr/tqag140

**Published:** 2026-06-05

**Authors:** Callum R T Kidson, Hyon-Mok Sohn, Joseph Page, Jonathan C L Rodrigues

**Affiliations:** Bristol Medical School, Faculty of Health & Life Sciences, University of Bristol, Bristol, BS8 1UD, United Kingdom; Department of Radiology, Royal United Hospitals Bath NHS Foundation Trust, Bath, BA1 3NG, United Kingdom; Respiratory Department, Royal United Hospitals Bath NHS Foundation Trust, Bath, BA1 3NG, United Kingdom; Department of Radiology, Royal United Hospitals Bath NHS Foundation Trust, Bath, BA1 3NG, United Kingdom; Department of Health, University of Bath, Bath, BA3 7AY, BA1 3NG, United Kingdom; The HLH Imaging Group LTD, Middlesex, HA4 0BW, United Kingdom

**Keywords:** computed tomography angiography, pulmonary embolism, incidental findings, clinical decision-making

## Abstract

**Objectives:**

Evaluate the prevalence of true-positive and false-positive incidental pulmonary embolism (iPE) diagnoses and the impact of expert review on diagnostic accuracy.

**Methods:**

This single-center retrospective study included consecutive contrast-enhanced thoracic CT examinations without dedicated PE protocols which reported iPE over an 18-month period (2022-24). Electronic health records were reviewed to identify requests to confirm PE based on previous non-dedicated studies. Studies identifying iPE were graded from 1 (most certain) to 5 by original report diagnostic certainty and compared between PE-positive and PE-negative subgroups using Fisher’s exact test. Identified imaging received retrospective expert review to calculate expert agreement with CTPA, where CTPA had been performed, using Cohen’s kappa.

**Results:**

Of 25 214 CT examinations, 260 (1%) reported iPE (mean age 73 ± 12 years; 51% women). Of these, 25% (*n = *65/260) underwent confirmatory CTPA. Patients receiving CTPA had lower initial iPE diagnostic certainty (median 3 versus 1, *P < .*01) and 52% (*n = *34/65) were PE-positive on CTPA. Patients with false-positive diagnoses had reduced diagnostic certainty on non-dedicated studies compared to those with a true-positive diagnosis (median 3 versus 2, *P < .*001). Agreement between the expert review of the iPE scan and the ground-truth CTPA diagnosis had κ of 0.96.

**Conclusions:**

Expert review could be an effective tool to reduce CTPA requirement in cases of intermediate (grade 3) iPE diagnostic certainty.

**Advances in knowledge:**

Low-certainty incidental pulmonary embolism diagnoses on initial non-dedicated CT are more likely to be false-positives according to CTPA findings or expert thoracic radiologist review. Expert review of initial CT may therefore reduce excessive imaging and avoid treating false-positive cases.

## Introduction

Venous thromboembolism, including pulmonary embolism (PE), is the third most common cardiovascular disease worldwide and is potentially life-threatening if not identified and treated promptly.^1^ In the United Kingdom, PE accounts for approximately 77 000 hospital presentations and 42 000 admissions annually.^2^ Although multidetector CTPA is the gold-standard imaging modality for PE diagnosis, the disease can be identified in contrast-enhanced CT (CECT) thoracic imaging without dedicated PE protocols with a reported prevalence of 0.7%-15% depending on the study cohort.^1^^,^^3^ This imaging is less sensitive due to suboptimal contrast phase and contrast mixing artifact which can mimic embolic defects.^4^ An observed rise in acute PE incidence may reflect broader increases in diagnostic imaging or increased recognition on non-dedicated imaging.^1^^,^^5^^,^^6^ These cases are termed incidental PE (iPE).

Current practice for confirming iPE lacks standardization.^5^ Therefore, clinicians may pursue confirmatory CTPA to clarify the diagnosis. Whilst this approach is more definitive, repeat imaging exposes patients to the risks conferred by additional ionizing radiation and intravenous contrast.^7^^,^^8^ In addition to these risks, repeat imaging imposes financial and opportunity costs on healthcare providers’ already strained imaging resources. Conversely, in cases without confirmatory CTPA follow-up where iPE is falsely diagnosed on non-dedicated imaging, there is inherent risk of overdiagnosis. Patients may receive unnecessary anticoagulation which confers well-documented risks.^9^

Therefore, improving the diagnostic accuracy of iPE through expert thoracic radiologist review may reduce harm from repeat imaging, minimize overtreatment, and reduce the burden of excess imaging. This approach could reduce both financial and opportunity costs for healthcare providers.

This single-centre retrospective study assesses the incidence of iPE across all non-dedicated CECT scans capturing the thorax. It also evaluates how frequently iPE prompted confirmatory CTPA imaging during routine care and assesses true-positive and false-negative diagnosis rates. The study assesses whether any patient characteristics, clinical factors or imaging-related features were associated with a false-positive iPE diagnosis on initial non-dedicated CT imaging.

The authors hypothesized that an expert thoracic radiology review could reduce requirements for confirmatory CTPA.

## Methods

### Ethics

This work was completed as a service evaluation with retrospective analysis of anonymized data obtained from routine care. Approval was granted by our hospital’s audit department. According to Health Research Authority standards, formal ethical approval was not required. Patients opted out of the national research scheme were excluded.

### Clinical cohort

All adult patients (≥18 years) undergoing CECT imaging at our hospital between December 1, 2022 and May 31, 2024 were screened for inclusion. Eligible scans included any CECT examination without a dedicated PE protocol that captured all, or part, of the patient’s thorax and reviewed by a radiologist in routine workflow. Studies performed with a concurrent CTPA, reporting previously diagnosed PE, or where PE was an indication for imaging, were excluded (Figure 1).

**Figure 1:**
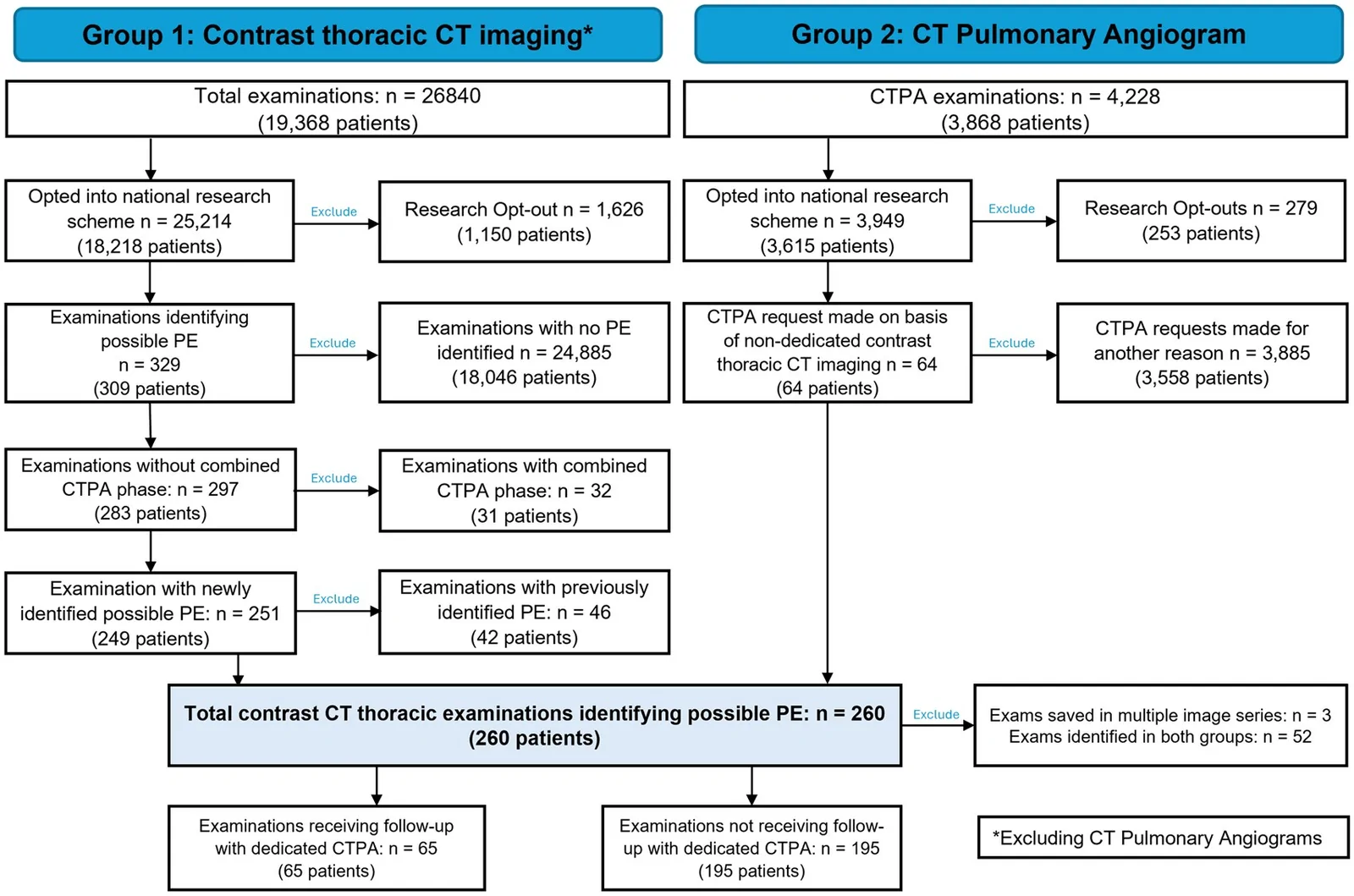
Flow diagram illustrating methods used to retrospectively identify patients with incidental PE on non-dedicated CT imaging and those receiving confirmatory CTPA. Two hundred sixty exams of unique patients were identified. Two methods identified 251 and 64 exams, respectively; 52 exams were identified by both methods, and 3 patients had a single exam saved in 2 image series.

The first method involved text searches of radiology reports using text strings to identify studies reporting iPE. To validate the search strategy (Table S1), a random 10% (*n = *2510) sample of reports were manually reviewed. This search strategy achieved a sensitivity and specificity of 100% and 84.0% (95% CI, 82.6-85.4), respectively. Within this sample, identification of iPE reporting was independently confirmed by a respiratory physician with 8 years clinical experience (J.P.), yielding an interobserver agreement of κ = 0.99 using Cohen’s κ. This approach identified 249 relevant patients.

To maximize this study’s sensitivity to imaging that received CTPA follow-up, all CTPA studies performed during the study period (*n = *3949) were identified separately and manually reviewed to identify those requested in response to previous non-dedicated CECT imaging that reported iPE (referred to as iCT). This approach identified 11 additional relevant patients.

### Certainty analysis of iCT reports

Original diagnostic certainty for iPE was assigned based on the language used in the original radiology report. This classification followed published guidance and used a 5-point scale, with grade 1 indicating the highest diagnostic certainty and grade 5 indicating the lowest (Table S2).^10^

### Expert review of iCT studies

All iCT imaging received an expert review by a cardiothoracic-fellowship-trained expert consultant radiologist with 10 years’ cardiothoracic subspecialty experience or a cardiothoracic fellow with 2 years’ cardiothoracic subspecialty experience as a reference standard. The reviewers were blinded to subsequent imaging. Each iCT study was classified as PE-positive, PE-negative, or indeterminate. Agreement between the expert’s iCT interpretation and the subsequent CTPA result was calculated excluding indeterminate cases from the analysis. Where the expert review was discrepant with CTPA imaging, a consensus review was carried out blinded to report outcome but with both iCT and CTPA studies available. Imaging without an iPE report was not reviewed.

### Clinical data

Median time intervals between the iCT and its initial report were recorded, as well as time to first identification of iPE in cases where it was not identified in the initial report. For patients who received confirmatory CTPA, anticoagulation status was documented both at the time of the iCT and at the time of the CTPA. Mortality data were also collected at 30 days and 1 year following the iCT. Mortality data were not corrected for confounders.

### Quantitative data

For iCT studies capturing the entire thorax, mean attenuation measurements of the main pulmonary artery (MPA), ascending aorta and descending thoracic aorta were obtained in a single axial slice. A region of interest was drawn within each vessel lumen to maximize cross-sectional area, while avoiding the vessel wall. The mean attenuation within each region of interest was recorded.

### CTPA acquisition

CTPA images were acquired using multidetector array scanners (SOMATOM Drive or Definition Edge, Siemens Healthineers, Erlangen, Germany). Acquisition used the following parameters: 145 kVP tube voltage and 120 mAs with automatic dose modulation, 1.2 pitch and 0.5 s rotation time with a slice thickness of 128 × 0.6 mm and acquisition matrix of 512 × 512. Nonionic intravenous contrast medium (iohexol, 350 mg iodine/mL; Omnipaque 350, Amersham Health, UK) was administered at a dose of 60 mL and a rate of 6 mL/s using an 18-G cannula. Images were acquired using bolus tracking, with a threshold of 100 HU in the MPA and a 4 s trigger delay.

### Statistics

Summary statistics are reported including age (mean ±SD) and sex distribution (% women). Differences in distribution of diagnostic certainty are compared using Fisher’s exact test and agreement is calculated using Cohen’s κ. The Kolmogorov-Smirnov test was used to assess for normality for quantitative CT data, and the Mann-Whitney *U* test is used to compare groups. Statistical analysis was performed using SPSS Statistics version 30 (IBM Corp.).

## Results

### Demographics

iPE was reported in 260 of the 25 214 studies, an incidence of 1.0%. The mean age of patients with iPE was 73 ± 12 years and 51% were women (Table 1). In 11 cases, iPE was identified in addenda from review of the imaging, having not been recognized in the initial report.

**Table 1:** Cohort demographics and study types.

	Whole cohort (*n = *260)	No CTPA follow-up (*n = *195)	CTPA follow-up (*n = *65)	
			PE-positive	PE-negative
Demographics				
Age, years (mean ±SD)	73 ± 12.3	74 ± 12.3	71 ± 11.1	67 ± 12.2
Sex, % women (*n*)	50.8 (132)	49.2 (96)	50.0 (17)	61.3 (19)
Study type, % (*n*)				
CT abdomen/pelvis with contrast	10.4 (27)	4.6 (9)	29.4 (10)	25.8 (8)
CT angio aortic arch and carotid Both	0.4 (1)	0 (0)	2.9 (1)	0 (0)
CT angiogram aorta	11.5 (30)	13.9 (27)	5.9 (2)	3.2 (1)
CT angiogram lower limb both	0.4 (1)	0.5 (1)	0 (0)	0 (0)
CT angiogram renal and abdominal	0.4 (1)	0.5 (1)	0 (0)	0 (0)
CT aorta whole with contrast	0.4 (1)	0.5 (1)	0 (0)	0 (0)
CT cardiac angiogram coronary	1.5 (4)	1.0 (2)	2.9 (1)	3.2 (1)
CT chest with contrast	11.2 (29)	11.8 (23)	8.8 (3)	9.7 (3)
CT chest/abdomen/pelvis with contrast	50.8 (132)	51.8 (101)	44.1 (15)	51.6 (16)
CT head neck thorax abdomen and pelvis	5.8 (15)	7.7 (15)	0 (0)	0 (0)
CT intravenous urogram	0.4 (1)	0 (0)	2.9 (1)	0 (0)
CT neck/thorax/abdomen/pelvis with contrast	6.5 (17)	7.7 (15)	0 (0)	6.5 (2)
CT pancreas with contrast	0.4 (1)	0 (0)	2.9 (1)	0 (0)

Of the 260 patients with reported iPE, 65 (25%) received layered CTPA testing to verify the diagnosis. This group had a mean age of 69 ± 12 years and 55% were women. Among these patients, 34 (52%) had a CTPA confirming the presence of acute PE (CTPA-positive) (Figure 2).

**Figure 2:**
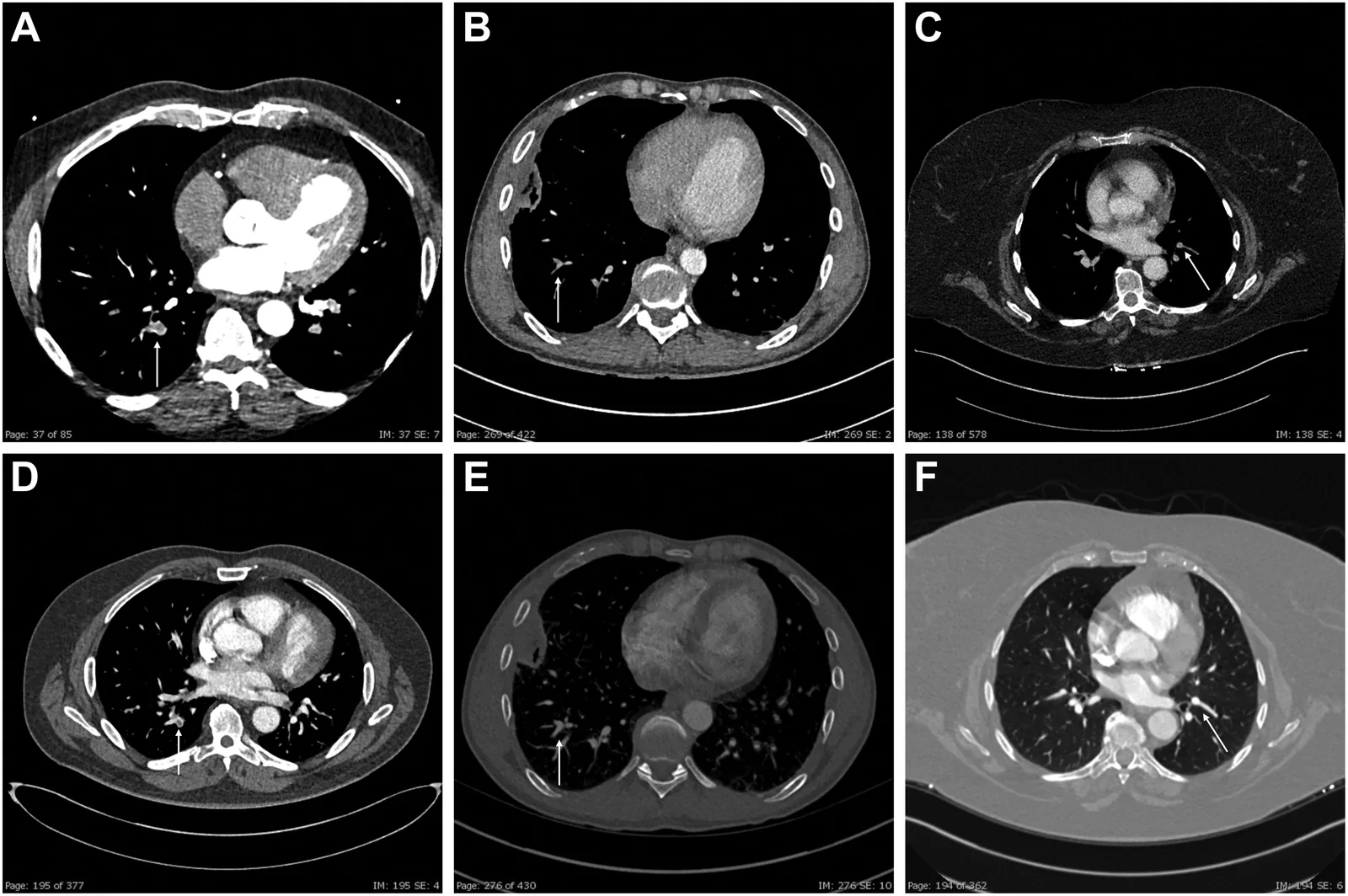
Non-dedicated imaging (A-C) and associated CTPA (D-F). Artifact/PE indicated by white arrow. (A) CT coronary angiogram with expert reporting suspected right lower lobe PE. (B) CT chest with expert report negative for PE. (C) CT chest, abdomen, and pelvis with expert report indeterminate for PE. (D) CTPA confirming presence of PE in A. (E) CTPA confirming no PE present in B. (F) CTPA demonstrating no PE in (C).

### Clinical features

The median time to first report for all iCT studies was 1.60 hours (IQR: 76.63 hours). The median time between iCT and confirmatory CTPA was 88.27 hours (IQR: 247.08 hours). The median time between PE identification and confirmatory CTPA was 45.57 hours (IQR: 114.65 hours) (Table 2).

**Table 2:** Time delays in clinical care of the overall cohort and, inpatient and outpatient subgroups.

Time delays, hours (median, IQR)	Whole cohort	No CTPA follow-up	CTPA follow-up		*P*
			PE-positive	PE-negative	
Inpatients (*n = *152)					
iCT to report	0.94, 0.73-1.33	0.93, 0.75-1.35	0.96, 0.59-1.25	1.03, 0.65-1.28	.119
iCT to PE report	0.99, 0.75-1.44	0.97, 0.75-1.40	1.03, 0.64-1.71	1.07, 0.82-2.02	.247
iCT to CTPA	22.28, 6.84-71.14	–	12.86, 6.15-37.14	44.65, 15.97-131.73	–
PE report to CTPA	15.65, 4.31-47.62	–	7.96, 3.15-18.97	47.24, 14.52-126.18	–
Outpatients (*n* = 108)					
iCT to report	98.75, 48.47-161.11	117.62, 65.58-163.38	62.8, 23.9-140.48	85.30, 48.80-155.73	.533
iCT to PE report	101.53, 48.79-165.55	118.57, 66.60-165.35	62.8, 23.9-140.48	94.51, 55.66-173.27	.626
iCT to CTPA	215.32, 108.44-373.36	–	192.73, 96.68-336.53	254.38, 146.33-389.82	–
PE report to CTPA	70.43, 24.83-216.43	–	70.43, 25.48-190.77	71.28, 29.23-216.48	–
Whole cohort (*n* = 260)					
iCT to report	1.60, 0.86-77.49	1.52, 0.82-91.66	1.97, 0.96-55.61	2.45, 1.02-76.63	.9
iCT to PE report	1.73, 0.91-93.40	1.58, 0.83-94.73	15.80, 1.025-66.825	14.17, 1.07-95.62	.353
iCT to CTPA	88.27, 23.78-270.87	–	46.43, 11.94-179.35	134.18, 30.83-315.52	–
PE report to CTPA	45.57, 13.57-128.22	–	24.18, 7.49-71.22	59.63, 23.35-204.66	–

*P* value compare group receiving versus not receiving CTPA follow-up.

Of the 34 CTPA-positive patients, one patient was already receiving treatment-dose anticoagulation for deep vein thrombosis, and 9 were on prophylactic doses before their iCT. Following iCT, 10 patients were receiving treatment-dose anticoagulation and 7 were receiving prophylactic doses. After PE confirmation on CTPA, all patients received treatment-dose anticoagulation.

Among the 31 patients with PE-negative CTPAs (CTPA-negative), 7 were receiving prophylactic-dose anticoagulation prior to their iCT and one was receiving a treatment dose for deep vein thrombosis. Following iCT, 11 patients received prophylactic doses of anticoagulation, and 2 received treatment-dose anticoagulation. In addition, one patient commenced treatment-dose anticoagulation for newly diagnosed atrial fibrillation. One patient was reported to have iPE which was later proven a false-positive on CTPA and thus was wrongly treated for PE.

High levels of mortality were observed in this cohort with 37/260 (14.2%) of patients dying within 30 days of iPE identification which rose to 95/260 (36.5%) after 1 year.

### Quantitative features

Across the whole cohort, there were 235 (90.4%) iCT studies that captured the entire thorax, thus allowing measurements of great vessel attenuation, of which 190 of 235 (80.9%) were in the group that received no CTPA follow-up and 45 (19.1%) were in the group receiving CTPA follow-up (21 PE-positive, 24 PE-negative) (Table 3).

**Table 3:** Attenuation data (where measurable) for great vessels divided by follow-up subgroups.

Attenuation, HU (median, IQR)	Whole cohort	No CTPA follow-up	CTPA follow-up	*P* value
Ascending aorta	198, 160-330	201, 161-333	183, 148-253	.08
Descending thoracic aorta	185, 148-304	188, 149-309	176, 137-248	.15
Main pulmonary artery	178, 150-291	189, 156-339	152, 125-183	<.001

### Certainty and expert review

Expert review of 65 cases that underwent confirmatory CTPA classified 25 (38.5%) cases as iPE-negative, 30 (46.2%) as iPE-positive, and 10 (15.4%) as indeterminate. In this group, 32 (49.2%) cases were classified as diagnostic certainty grade 1 or 2 (highest certainty), 29 (44.6%) as grade 3 and 4 (6.2%) as grade 4 or 5.

There was a significant difference in the distribution of diagnostic certainty between the CTPA-positive and CTPA-negative groups (*P < .*01). Among patients with an iPE diagnosis later confirmed as CTPA-positive, 23 of 34 (67.6%) were initially classified as diagnostic certainty grade 1 or 2. In contrast, only 9 out of 31 (29.0%) CTPA-negative cases were assigned these higher grades (Figure 3, Table S3).

**Figure 3:**
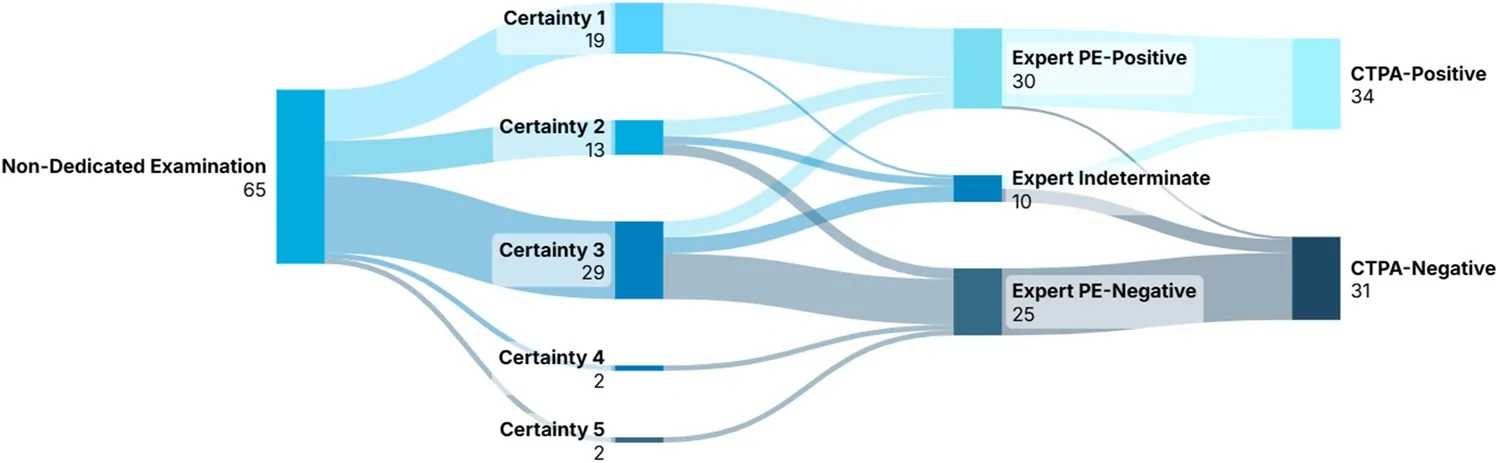
Sankey diagram showing the original diagnostic certainty of non-dedicated CT identifying PE receiving CTPA follow-up, and the subsequent consensus expert and CTPA classification of these diagnoses.

Expert review of cases that did not receive follow-up CTPA identified the majority (*n = *177/194, 91.2%) as iPE-positive with 5 (2.6%) deemed iPE-negative and 12 (6.2%) indeterminate. In this group, 177 (91.2%) were classified as diagnostic certainty grade 1 or 2, 10 (5.2%) as grade 3 and 7 (3.6%) as grade 4 or 5 (Figure 4). A patient in this group with an iPE diagnostic certainty grade of 3 was missing images associated with the reported iPE and thus could not be reviewed. There was a significant difference in the distribution of diagnostic certainty between the iCT scans that received CTPA follow-up versus those that did not (*P < .*001).

**Figure 4:**
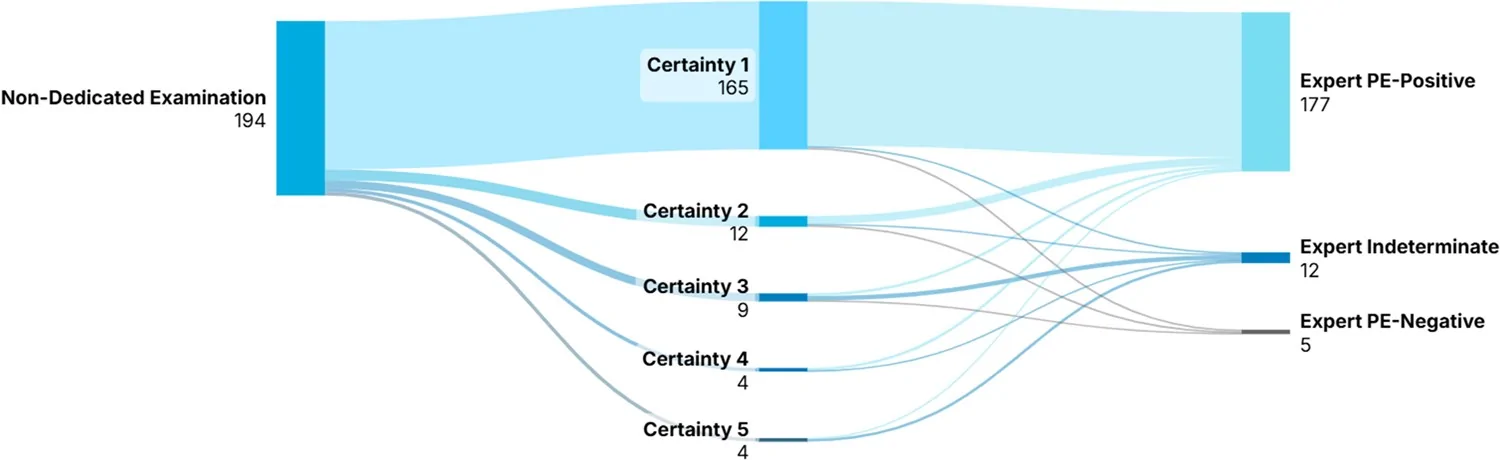
Sankey diagram showing the diagnostic certainty of non-dedicated CT identifying PE not receiving CTPA follow-up, and expert review of these diagnoses.

Before arbitration, the agreement between the expert and subsequent CTPA report was 0.78, excluding indeterminate cases. Initial expert review correctly predicted 27 cases that were CTPA-positive and 22 cases that were CTPA-negative. There were 10 cases deemed indeterminate and 6 incorrectly categorized with 3 CTPA-negative iCT studies classified as iPE-positive and 3 CTPA-positive iCT studies classified as iPE-negative. Following arbitration and review of CTPA, 2 cases initially reported as expert-negative were reclassified as positive and one revealed a PE falsely reported on CTPA. This resulted in an agreement between expert review and CTPA of 0.96. One case remained discrepant with a CTPA-negative iCT classified as iPE-positive (Figure 3).

## Discussion

To the best of the authors’ knowledge, this is the first study to assess the prevalence of iPE with concurrent consideration of confirmatory CTPA and dedicated expert review of all iPE cases. The findings indicate that iPE is reported in 1% of all CECT, with 25% of these cases receiving confirmatory CTPA. In this subset, the false-positive rate was 46% and the original diagnostic certainty was lower compared to the 75% that did not receive confirmatory CTPA (*P < .*001). These findings suggest that low diagnostic certainty of iPE in routine radiology workflows is associated with a spurious diagnosis.

Current clinical practice lacks formal guidance on confirmatory CTPA consideration following an iPE diagnosis with its use at the discretion of the responsible clinician. These findings suggest that diagnostic certainty may strongly influence that decision, as a greater proportion of iPE cases with diagnostic certainty grades 1 or 2 did not progress to confirmatory CTPA. This observation may additionally validate the method of certainty grading used for analysis. Among those who received confirmatory CTPA, only 52% were CTPA-positive, highlighting the need to evaluate how radiological diagnosis of iPE influences subsequent investigations and management.

A key consideration is whether the initial iPE diagnosis was appropriate given the image quality of the CECT. There is no agreed threshold for MPA attenuation for a diagnostic CTPA, but widely accepted thresholds are from 210 to 250 HU.^11^^,^^12^ Among iCT studies where MPA attenuation was measurable, 37.5% (88/235) met the lower threshold, including 17.8% (8/45) that were followed up with confirmatory CTPA. Importantly, no studies demonstrated MPA attenuation below the theoretical minimum threshold of 93 HU for acute PE detection.^13^ This suggests that all studies were potentially adequate to identify iPE. The noted significance (*P < .*001) of the difference in MPA attenuation values between the group receiving CTPA follow-up and the group that did not potentially reflects the increased quality of scan in the no follow-up group increasing diagnostic certainty.

Expert thoracic radiologist review classified 46.2% (30/65) of iCTs with confirmatory CTPA as iPE-positive, compared with 91.2% (177/194) in those who did not receive confirmatory CTPA. The high level of predictive agreement between expert review and CTPA outcome provides reassurance that iPE diagnoses not subjected to CTPA were likely accurate. A revised workflow could help reduce the risks associated with additional contrast, radiation exposure, and unnecessary anticoagulation.^7–9^ Limiting the use of confirmatory CTPA may lessen financial burden and reclaim opportunity costs for healthcare providers.

The agreement between expert review and CTPA was substantial before consensus reading (κ = 0.78) and excellent following a consensus reading (κ = 0.96), as one might wish for in a healthcare setting.^14^ Notably, the one case of disagreement between expert iCT interpretation and CTPA reporting had a delay between iCT and CTPA of 14 days and received treatment-dose anticoagulation for a segmental PE, potentially accounting for this disagreement.

Given this context, an alternative iPE workflow warrants consideration. The use of a predefined lexicon at the time of reporting a CECT could facilitate improved risk stratification when iPE is identified. High-certainty cases could proceed with standard care, while intermediate-certainty (grade 3) cases could be referred for dedicated, expert thoracic radiology review to guide further management.

Although this proposed workflow would require access to subspecialist thoracic radiologists, it may be justified given the predictive value of diagnostic certainty and the high proportion of false positives among intermediate-grade cases. Notably, all patients with diagnostic certainty grades below 3 were PE-negative at confirmatory CTPA, raising questions regarding the added value of further testing in low-certainty cases. This approach could alleviate demand on radiology services by reducing imaging requirements.

It is important to note the high mortality rates in both patients receiving and not receiving confirmatory CTPA. Data on the cause of death were not universally available via hospital electronic health records, however, where documented, deaths were often due to metastatic malignancy which was the primary indication for the patients’ initial CTs. This is relevant to real-world clinical practice and further highlights the importance of contextual clinical judgment in managing iPE and supports minimizing unnecessary investigations in patients with advanced disease.

### Limitations

This study has several limitations. Firstly, the retrospective nature of this study is limited by the complexity and variability that occurs within clinical decision-making and a lack of study control over selection of patients to receive CTPA potentially contributing to selection bias. Delays between iCT and confirmatory CTPA varied widely and may have influenced study results. Reasons for delay are difficult to reconstruct or represent quantitatively. Additionally, imaging where radiologists had not reported iPE was not reviewed for the purposes of this study and, as such, there is no available data on occurrence of missed iPE.

Furthermore, it is unclear why some patients with high diagnostic certainty for iPE received a confirmatory CTPA. One explanation could be that CTPA was used to assess thrombus burden rather than confirm PE diagnosis. However, this distinction is not consistently clear in clinical records. Conversely, it is also unclear why some low certainty diagnoses did not receive confirmatory CTPA. This likely reflects heterogeneity in clinical decision-making that contributed to 195 patients not receiving confirmatory CTPA.

Radiological reporting is renowned for inter-reporter variability when communicating diagnostic certainty which further limits analysis and implementation of a certainty-based workflow.^4^ Standardized reporting of diagnostic certainty may help overcome this issue and improve reproducibility.^10^ Differences in reporting practices between in-house radiologists and those from outsourced services, for whom thoracic radiology expertise is unknown, may also have introduced bias. Finally, iPE is often underreported and the true prevalence of iPE may be underestimated.^3^

## Conclusion

Incidental PE was reported in 1% of all CECT scans within this single-center study. Just over half (52%) of cases followed up by CTPA were PE-positive. In patients not receiving CTPA, the false-positive rate was likely lower given the higher diagnostic certainty at initial reporting. The study findings suggest that incorporating a certainty-based workflow could have reduced layered testing by 85% while maintaining diagnostic accuracy. This strategy could reduce reliance on confirmatory CTPA; alleviating pressure on radiology departments, reducing costs and minimizing patient exposure to unnecessary contrast and radiation. These findings highlight a need for prospective validation of a certainty-stratified approach to iPE, where expert review of cases with diagnostic uncertainty may enhance accuracy and reduce unnecessary imaging.

## Supplementary Material

tqag140_Supplementary_Data
