# Supplementary material for: Incidental pulmonary embolism and the value of CT pulmonary angiography follow-up versus expert review
Source: Br J Radiol. 2026 Jun 5;99(1186):2164–71. doi: 10.1093/bjr/tqag140 (PMC13623241; doi:10.1093/bjr/tqag140)
Supplement: Supplementary file 1 [file tqag140_supplementary_data.zip › Supplementary Tables.docx]

**Supplementary Tables**

| **Inclusion** | **Exclusion** | **Override** |
| --- | --- | --- |
| embolism | no visible pulmonary emboli | CTPA for definitive |
| thromboembolism | no signs of pulmonary emboli | pulmonary angiogram for definitive |
| embolus | PE0 |  |
| emboli | no incidental pulmonary emboli |  |
| thrombus | no CT evidence of pulmonary emboli |  |
| thromboemboli | no evidence of pulmonary emboli |  |
| PE1 | no gross incidental pulmonary emboli |  |
| PE=1 | No pulmonary artery filling defect |  |
| thromboembolus | No PE |  |
| PE2 | No PE. |  |
| PE=2 | no visible pulmonary embolus |  |
| PE | no visible PE |  |
| ?PE | no visible PE. |  |
| PE. | no signs of pulmonary embolus |  |
| filling defect | no signs of PE |  |
|  | no signs of PE. |  |
|  | no incidental pulmonary embolus |  |
|  | no incidental PE |  |
|  | no incidental PE. |  |
|  | no CT evidence of pulmonary embolus |  |
|  | no CT evidence of PE |  |
|  | no CT evidence of PE. |  |
|  | no evidence of pulmonary embolus |  |
|  | no evidence of PE |  |
|  | no evidence of PE. |  |
|  | no gross incidental pulmonary embolus |  |
|  | no gross incidental PE |  |
|  | no gross incidental PE. |  |

***Table S1:*** *Phrases used for a text search of CT report records using a formula in Excel 365 (Microsoft). Reports were assigned a score of +1 for containing a phrase in the Inclusion column, -1 for a phrase in the Exclusion column and +1 for a phrase in the Override column. Reports with a score >0 were manually reviewed. The Exclusion phrases were used to raise search specificity.*

| **Certainty** | **Associated Language** |
| --- | --- |
| *1* | there is^a^, highly suggestive, suggests, strong suspicion, consistent with, in keeping with |
| *2* | concern for, suspicion, probable, impression, could suggest |
| *3* | equivocal, mildly concerning for, could represent, potential, possible |
| *4* | likely artefactual, indeterminate but suspicious |
| *5* | unlikely, doubtful significance, cannot exclude, cannot rule out |
| ^a^ including statement of diagnosis (eg, "Multiple pulmonary emboli") | |

***Table S2:*** *Language identified in radiological reports organised according to diagnostic certainty.^10^*

| **Diagnostic Certainty of Original iCT Report** | **No CTPA Follow-up (n)** | | | **CTPA Follow-up (n)** | | | | |
| --- | --- | --- | --- | --- | --- | --- | --- | --- |
|  | **Expert Review** | | | **Expert Review** | | | **CTPA** | |
|  | ***PE Positive*** | ***PE Negative*** | ***Indeterminate*** | ***PE Positive*** | ***PE Negative*** | ***Indeterminate*** | ***PE*** | ***No PE*** |
| *1* | 161 | 2 | 2 | 18 | 0 | 1 | 16 | 3 |
| *2* | 9 | 2 | 1 | 6 | 4 | 3 | 7 | 6 |
| *3* | 4 | 1 | 5 | 5 | 16 | 8 | 11 | 18 |
| *4* | 3 | 0 | 1 | 0 | 2 | 0 | 0 | 2 |
| *5* | 1 | 0 | 2 | 0 | 2 | 0 | 0 | 2 |
| ***Total*** | 178 | 5 | 11 | 29 | 24 | 12 | 34 | 31 |

***Table S3:*** *PE status stratified by diagnostic certainty of the radiological report of the original non-dedicated contrast CT incidentally identifying PE according to expert review and CTPA.*
